# Intercellular Adhesion Molecule 1: More than a Leukocyte Adhesion Molecule

**DOI:** 10.3390/biology12050743

**Published:** 2023-05-19

**Authors:** Cameron D. Haydinger, Liam M. Ashander, Alwin Chun Rong Tan, Justine R. Smith

**Affiliations:** College of Medicine and Public Health, Flinders University, Adelaide, SA 5042, Australia; cameron.haydinger@flinders.edu.au (C.D.H.); liam.ashander@flinders.edu.au (L.M.A.); alwintan1994@gmail.com (A.C.R.T.)

**Keywords:** intercellular adhesion molecule 1, ICAM-1, immune system, inflammation, infection, cancer, atherosclerosis

## Abstract

**Simple Summary:**

Intercellular adhesion molecule 1 (ICAM-1) is a protein produced by cells in blood vessel walls, as well as many other cell types. It localizes to the surface of these cells to allow them to communicate with adjacent cells. The production of ICAM-1 is induced by inflammation, such as occurs during infection, when one of its main functions is to direct white blood cells to the site of the infection. However, in autoimmune diseases and certain types of infection, ICAM-1 can be involved in directing cells to damage healthy tissue. ICAM-1 can also promote or protect against cancer, depending on the type of cancer and the cell populations that are producing it. In this review article, the *ICAM1* gene and ICAM-1 protein are introduced, and their regulation is summarized. The roles of ICAM-1 in the immune system and a spectrum of diseases, as well as therapeutics that target ICAM-1, are discussed.

**Abstract:**

Intercellular adhesion molecule 1 (ICAM-1) is a transmembrane protein in the immunoglobulin superfamily expressed on the surface of multiple cell populations and upregulated by inflammatory stimuli. It mediates cellular adhesive interactions by binding to the β2 integrins macrophage antigen 1 and leukocyte function-associated antigen 1, as well as other ligands. It has important roles in the immune system, including in leukocyte adhesion to the endothelium and transendothelial migration, and at the immunological synapse formed between lymphocytes and antigen-presenting cells. ICAM-1 has also been implicated in the pathophysiology of diverse diseases from cardiovascular diseases to autoimmune disorders, certain infections, and cancer. In this review, we summarize the current understanding of the structure and regulation of the *ICAM1* gene and the ICAM-1 protein. We discuss the roles of ICAM-1 in the normal immune system and a selection of diseases to highlight the breadth and often double-edged nature of its functions. Finally, we discuss current therapeutics and opportunities for advancements.

## 1. Introduction

Intercellular adhesion molecule 1 (ICAM-1) is an immunoglobulin (Ig) superfamily protein expressed on many different cell types including endothelial cells, epithelial cells, fibroblasts, and leukocytes [1]. The ICAM-1 promoter contains binding sites for numerous transcription factors including activator protein 1 (AP-1) (Jun/Fos), specificity protein 1 (SP1), nuclear factor kappa B (NF-κB), interferon regulatory factor 1 (IRF1), IRF3, and ETS1 [2,3,4,5,6]. Many cells exhibit basal expression of ICAM-1, which is highly upregulated by a wide variety of inflammatory stimuli, including the cytokines tumor necrosis factor alpha (TNF-α), interleukin 1 beta (IL-1β) [7], interferon gamma (IFN-γ), and interleukin 6 (IL-6) [8], as well as reactive oxygen species [6], high glucose [9], and shear stress [10]. Major signaling pathways involved in the upregulation of ICAM-1 transcription in response to various stimuli include NF-κB, mitogen activated protein kinases (MAPKs), and Janus kinase-signal transducer and activator of transcription (JAK-STAT) [2]. The regulation of ICAM-1 expression takes place largely at the level of transcription and is highly cell-type- and stimulus-specific [11].

ICAM-1 acts a receptor for the β2 integrins macrophage-1 antigen (MAC-1) [12] and lymphocyte function-associated antigen 1 (LFA-1) [13], hyaluronan [14], fibrinogen [15], major group rhinoviruses [16], and particular proteins encoded by the apicomplexan parasites *Plasmodium falciparum* [17] and *Toxoplasma gondii* [18]. ICAM-1 participates in homeostatic immune responses that extend from the mediation of leukocyte trafficking [19] to the formation of the immune synapse [20], and to the healing of tissue wounds [21]. Increased expression of the protein has been implicated in pathological inflammation in numerous diseases including sepsis [22], autoimmune disorders [23], cancer [24], and cardiovascular conditions [25]. Given its central role in mediating leukocyte extravasation and involvement in a diverse suite of diseases, ICAM-1 has long been a tempting therapeutic target in pathological inflammation. To date, few therapeutics have been successful, although a more complete understanding of the molecular mechanisms controlling ICAM-1 expression and more precise methods of targeting treatment may soon shift the balance in our favor. Here we review the literature on ICAM-1 with a particular focus on pathological inflammation and the potential for new targeted therapies.

## 2. *ICAM1* Gene

### 2.1. Gene Structure and Regulation of Transcription

The human *ICAM1* gene is located on the short arm of chromosome 19 (19p13.2), in an 80 kb gene cluster also containing gene family members *ICAM3*, *ICAM4*, and *ICAM5* [26]. The remaining gene of the ICAM family, *ICAM2*, has been mapped to chromosome 17 [27]. The *ICAM1* gene is comprised of seven exons and six introns [3].

Early work in mapping the 5′-regulatory region of the *ICAM1* gene identified a number of key promoter elements driving ICAM-1 upregulation in response to immunological stimuli [3]. Based on work using human umbilical vein endothelial cells (HUVECs), the increase in gene transcription in response to TNF-α and IL-1β was originally thought to be driven solely by binding of p65 (RELA) homodimers or p65/p50 (NFKB1) heterodimers to an NF-κB binding site located between −187 and −178 base pairs (bp) upstream of the translation start site, as mutation of this binding sequence abolished the *ICAM1* gene response to TNF-α stimulation [7]. The identification of a CCAAT/enhancer binding protein (C/EBP) element adjacent to this NF-κB binding sequence, and the discovery that mutation of the C/EBP binding sequence reduced but did not eliminate TNF-α-mediated increase in ICAM-1, indicated that both NF-κB and the enhancer were required for full induction of *ICAM1* gene transcription [28]. Working in mouse models of endothelial activation, Lv et al. showed a role for the transcriptional coactivator, yes-associated protein 1 (YAP1), as a checkpoint modulator of ICAM-1 transcription [29].

In the case of TNF-α, evidence long suggested that NF-κB was the primary driver of ICAM-1 upregulation to the exclusion of other transcription factors [30]. Newly published work, however, suggests that while NF-κB undoubtedly drives early increases in ICAM-1 and other inflammatory transcripts, the JAK/STAT pathway may be more important in maintaining the endothelium in a chronic inflammatory state after the cytokine stimulus is removed [31]. Other recent research has implicated the protein tyrosine kinases focal adhesion kinase (FAK) and proline-rich tyrosine kinase 2 (Pyk2) in the upregulation of ICAM-1 in response to both TNF-α and IL-1β [32]. Pharmacological and siRNA-mediated inhibition of FAK/Pyk2 blocked the activation of c-Jun N-terminal kinase (JNK) and extracellular signal-regulated kinase (ERK) and reduced ICAM-1 transcript and protein levels in human aortic endothelial cells. Interestingly, the phosphorylation of NF-κB p65 was largely unchanged, suggesting the involvement of other transcription factors. The role of the transcription factor IRF3 in ICAM-1 induction in HUVECs has also been studied, with Liu et al. demonstrating direct binding of IRF3 to the *ICAM1* promoter, while Zhu et al. have shown that IRF3-dependent increase in ICAM-1 is mediated by activation of Akt1 in the context of low shear stress [5,33].

The upregulation of ICAM-1 in response to IFN-γ is driven by the binding of STAT1 homodimers to an IFN-γ response element located in a region of the promoter −116 to −36 bp upstream of the translation start site of the *ICAM1* gene [34,35]. The induction of ICAM-1 by IL-6 involves the binding of phosphorylated STAT3, as a homodimer or heterodimer with STAT1, to a promoter element in this same region [8,36].

### 2.2. Alternative Transcripts

**Figure 1:**
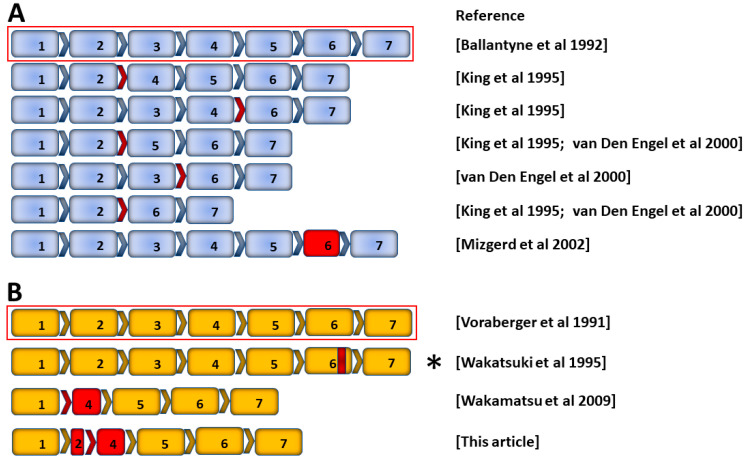
Schematic of ICAM-1 mRNA and alternative transcripts in mouse and human. Exon arrangement of published full-length and alternative transcripts from (**A**) mouse *Icam1* and (**B**) human *ICAM1* genes [3,37,38,39,41,43,44]. Full-length ICAM-1 transcripts encoding transmembrane ICAM-1 protein are indicated by red outlines, above transcripts arising from alternative splicing or deletions. Red arrows denote exon skipping, and solid red boxes denote truncated exons. Asterisk identifies a transcript encoding human soluble ICAM-1 protein: partial deletion of exon 6 (red band) results in a frame shift and early termination of translation, producing a protein that lacks the transmembrane and cytoplasmic domains.

Alternative splicing events generating functional ICAM-1 protein isoforms are well described in the mouse (Figure 1A) [37,38,39]. Most murine ICAM-1 isoforms are generated through exon skipping [39]. Less work has been done to identify alternatively spliced human transcripts, although murine and human ICAM-1 display a high degree of homology (Figure 1B) [40,41]. The *Homo sapiens ICAM1* gene entry for Ensembl (ENSG00000090339, release 109 [42]) lists two transcripts with complete protein-coding sequences, the full-length ICAM-1 transcript (ENST00000264832.8) and one alternative transcript (ENST00000423829.2) that was identified in human synovial membranes as part of a large-scale sequencing project [43]. In addition, a soluble isoform is generated through alternate splicing (see Section 3.5) [44].

**Figure 2:**
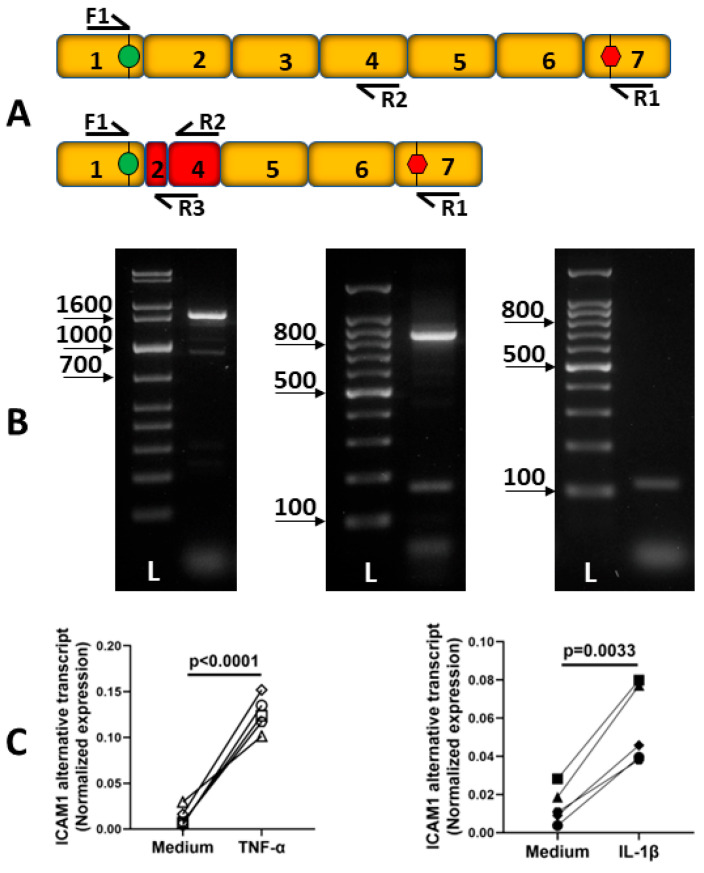
Description of human ICAM-1 alternative transcript. (**A**) Schematic indicating the location of polymerase chain reaction (PCR) primers on full-length and alternatively spliced ICAM-1 transcripts. F1: 5′-CAACCTCAGCCTCGCTATGG-3′, R1: 5′-TCAGGGAGGCGTGGCTTGTGTGTT-3′, R2: 5′-TCACACTGACTGAGGCCTT-3′, R3: 5′-TGGGGTTCAACCTCTGGTCATT-3′. (**B**) Three 2% agarose gel images showing ICAM-1 RT-PCR products amplified from human retinal endothelial cell cDNA using the F1/R1 primer set (panel 1, expected product size: full-length transcript = 1614 bp, alternative transcript = 928 bp), the F1/R2 primer set (panel 2, expected product size: full-length transcript = 851 bp, alternative transcript = 164 bp), and the F1/R3 primer set (panel 3, expected product size: full-length transcript = no product, alternative transcript = 113 bp). L = DNA ladder: 1 kbp (panel 1) or 100 bp (panels 2 and 3). (**C**) Expression of human ICAM-1 alternative transcript in primary human retinal endothelial cell isolates from 10 individual donors following 4 h of treatment with TNF-α 10 ng/mL (panel 1; *n* = 5 human isolates) or IL-1β 5 ng/mL (panel 2; *n* = 5 human isolates) measured by real-time quantitative PCR of cDNA (F1 and R3 primers). The data were analyzed by unpaired Student’s *t*-test.

Previously unpublished work from our laboratory suggests the existence of another alternatively spliced human ICAM-1 transcript (Figure 2A,B). Sequencing indicates alternative splicing between exons 2 and 4, resulting in a 912 bp predicted coding sequence that excludes all but 12 bp of exon 2 plus the entire exon 3, and contains a truncated exon 4. Splicing between exons 2 and 4 results in an in-frame transcript, and translation predicts a 303 amino acid peptide matching ICAM-1 except for a single substitution, A27D, occurring at the end of the predicted N-terminal signal peptide. This alternative transcript is inducible by TNF-α and IL-1β (Figure 2C).

### 2.3. Post-Transcriptional Regulation

Stabilization of mRNA has been implicated in the induction of ICAM-1 observed under different conditions. Ohh et al. observed a significant increase in the half-life of ICAM-1 mRNA following phorbol myristate acetate or IFN-γ treatment of the mouse monocytic cell line P388D1 or fibroblast L cells transfected to express ICAM-1 [45]. The stabilization of ICAM-1 mRNA by phorbol myristate acetate, but not IFN-γ, was dependent on AUUUA motifs in the ICAM-1 mRNA 3′-untranslated region [45]. Shi et al. demonstrated that siRNA knockdown of MAPK-activated protein kinase 2 (MAPKAPK2) prevented inactivation of the RNA degradation mediator tristetraprolin after human lung microvascular endothelial cells were treated with TNF-α, promoting degradation of ICAM-1 mRNA [46]. Further study showed that siRNA knockdown of MAPKAPK2 resulted in reduced cytoplasmic accumulation of the RNA binding protein human antigen R (HuR), which stabilized mRNA transcripts by binding to AU-rich elements. HuR knockdown decreased ICAM-1 mRNA half-life, ICAM-1 protein levels, and binding of neutrophils to endothelial monolayers [47]. Taken together, these results indicate that, at least in lung microvascular endothelial cells, TNF-α stimulation prolongs ICAM-1 mRNA half-life by both inhibiting degradation and increasing mRNA stability in a MAPKAPK2-dependent manner. Whether this mechanism is generalizable to other endothelial cell types is unknown.

Growing interest in regulation by non-coding regulatory RNA has led to many studies investigating the post-transcriptional regulation of ICAM-1, most focusing on microRNA (miRNA). Using target prediction algorithms, Suárez et al. identified putative binding sites for miR-17-3p in the 3′-untranslated region of the ICAM-1 transcript [48]. In HUVECs, miR-17-3p repressed luciferase reporter activity of the ICAM-1 3′-untranslated region and overexpression of this microRNA reduced neutrophil binding after TNF-α stimulation. As the expression of miR-17-3p is itself induced by TNF-α via NF-κB, the study provided an elegant demonstration of ICAM-1 post-transcriptional regulation by miRNA-mediated negative feedback. Additional studies have implicated numerous regulatory RNAs in the post-transcriptional regulation of ICAM-1 expression in various contexts, including miR-126-3p in the context of IL-6 stimulation [49]; miR-150-5p in acute respiratory distress syndrome [50] and allergic rhinitis [51], miR-223 in Kawasaki disease [52,53] and sepsis [54], miR148b-3p in coronary artery disease [55], and ICAM-1 related long non-coding RNA (ICR) in atherosclerosis [56], retinal vasculopathy [57], and hepatocellular carcinoma [58].

### 2.4. Icam1 Gene-Deficient Mouse Models

Prior to the awareness of alternative ICAM-1 isoforms, two different mouse strains were generated by insertion of a neomycin resistance cassette into a target exon within the *Icam1* coding region [59,60]. A result of alternative splicing, this strategy unintentionally permitted the residual expression of isoforms lacking the targeted exon, and although the issue was quickly recognized [37], these strains have been widely used with results often presented assuming complete *Icam1* gene knockout. Importantly, the strains do not express the full-length protein, and work using them provides valuable information; however, there are instances of phenotypic differences between these strains and more recently generated complete *Icam1* gene knockouts, so the existence of alternative isoforms should be borne in mind when interpreting the literature. More information is summarized in a review by Ramos et al. [61]. In terms of nomenclature, the *Icam1^tm1Bay^* mouse contains an insert in exon 5 [60], the *Icam1^tm1Jcgr^* mouse contains an insert in exon 4 [59], and *Icam1^null^* refers to a mouse lacking all ICAM-1 isoforms. In the present review, the designation *Icam1^KO?^* is applied where the mutant mouse strain used in a study is not clear.

## 3. ICAM-1 Protein

### 3.1. Protein Structure

ICAM-1 is a member of the Ig superfamily of transmembrane proteins. It is a type I transmembrane protein with a peptide core of 55 kilodaltons. The mature protein is heavily glycosylated, 76–114 kilodaltons, depending on modifications [1]. The membrane-bound isoform is composed of five extracellular Ig domains (D1 through D5), a transmembrane domain, and a short cytoplasmic tail that interacts with the actin cytoskeleton. The Ig domains are arranged end to end with stabilizing disulfide bonds at conserved cysteine residues [13]. There are five known members of the ICAM protein family [62], ICAM-1 shares a high degree of sequence homology with ICAM-3 and ICAM-5, composed of five and nine Ig domains, respectively. ICAM-1 and ICAM-2 share the most functional redundancy; both molecules are expressed on endothelial cells and have distinct but overlapping roles [63,64,65].

ICAM-1 binds LFA-1 through a highly conserved glutamic acid residue (Glu34) in D1, while conserved aspartic acid (Asp229) and glutamic acid (Glu254) residues in D3 mediate ICAM-1 binding to MAC-1 [66]. Research by Miller et al. and Reilly et al. suggested that native ICAM-1 protein formed homodimers both as the soluble form and on the cell surface and displayed an increased affinity for LFA-1 when dimerized [67,68]. Crystal structure modelling indicates that ICAM-1 forms homodimers through the interaction of D3 through D5, with D4 containing the critical region required for dimerization. Dimerization at D4, combined with a characteristic bend between D3 and D4, is thought to position the critical residues optimally for ligand binding [69].

At least six membrane-bound isoforms and one soluble isoform of ICAM-1 have been described, each containing a variable number of Ig domains with corresponding differences in the ability to bind LFA-1. While functional protein isoforms lacking certain Ig domains, arising from alternatively spliced transcripts, have been identified and characterized in the mouse [61], we are not aware of any studies demonstrating the presence of functional membrane-bound ICAM-1 isoforms in humans.

### 3.2. Post-Translational Modification

Human ICAM-1 can be post- or co-translationally modified by N-glycosylation at eight sites with functional consequences for ligand binding [70,71]. More recently, studies of a hypoglycosylated high-mannose ICAM-1 modification and the functional implications for leukocyte recruitment have been investigated. Scott et al. reported the presence of high-mannose ICAM-1 in human coronary arteries, indicating that hypoglycosylated forms of ICAM-1 are present in vivo [72]. They further demonstrated that the hypoglycosylated high-mannose ICAM-1 was functional, supporting monocyte adhesion and cell signaling. Regal-McDonald et al. observed transient expression of high-mannose ICAM-1 following TNF-α stimulation, and demonstrated that high-mannose ICAM-1 on the surface of HUVECs or COS-1 cells expressing human ICAM-1 selectively increased the adhesion of CD16-positive monocytes [73].

### 3.3. ICAM-1 Ligands

The primary ligands of ICAM-1 are the β2 integrins MAC-1, a heterodimer of CD18 and CD11b (also known as αMβ2), and LFA-1, a heterodimer of CD11a and CD18 (also known as αLβ2). All leukocyte subsets express at least one of these β2 integrins [74]. LFA-1 exists in multiple conformational states, with the activated conformation binding ICAM-1 with the highest affinity [75]. Switching between conformations is mediated by integrin-adhesion molecule interactions as well as chemokine signaling [76]. ICAM-1 binding occurs via interaction with a metal ion-dependent adhesion site (MIDAS), located in the αL Inserted (I) domain of LFA-1. When LFA-1 is activated, the magnesium ion contained within the MIDAS is positioned optimally to bind the Glu34 residue in ICAM-1 D1, forming a high-affinity bond stabilized by multiple hydrogen bonds and salt bridges [75,77]. A structurally similar I domain has been described for MAC-1, although the low- and high-affinity ICAM-1 binding conformational states differ between the two integrins [78,79].

ICAM-1 can bind other ligands, such as hyaluronan and fibrinogen. Numerous studies have addressed the roles of these alternative ligands, particularly fibrinogen, as mediators of ICAM-1-leukocyte binding interactions and activators of intracellular signaling cascades [14,15,80]. Recently, Sulimai et al. demonstrated a direct role for ICAM-1 signaling triggered by binding to fibrinogen in the NF-κB-mediated up-regulation of pro-inflammatory cytokines by primary mouse cortical neurons [81].

### 3.4. Intracellular Signaling

**Figure 3:**
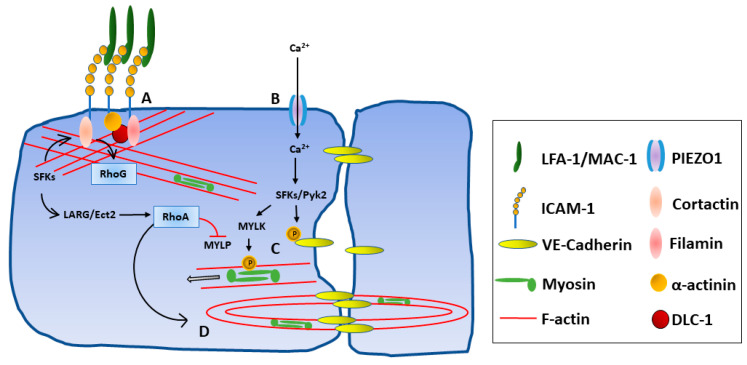
ICAM-1 intracellular signaling after integrin binding. Key proteins involved in intracellular signaling during leukocyte migration interactions are shown, noting that pathways continue under study. (A) Integrin binding enhances ICAM-1 clustering and actin cytoskeleton remodeling via the recruitment of numerous adaptor proteins (e.g., cortactin, α-actinin, and filamin) and activation of guanidine nucleotide exchange factors (e.g., Ect2 and LARG) and small GTPases (e.g., RhoA and RhoG). (B) Endothelial membrane tension is increased, triggering opening of mechanosensitive cation channels (e.g., PIEZO1) and an increase in intracellular calcium ion. (C) Increased calcium ion leads to phosphorylation of VE-Cadherin by Src-family kinases (SFKs) and Pyk2 and dissociation of adherens junctions. Phosphorylation of myosin light chain by myosin light chain kinase (MYLK) and the resulting actin-myosin contraction opens cell–cell junctions. (D) The RhoA-dependent formation of F-actin contractile pores is mediated in part by activation of LARG and Ect2.

In endothelial cells, intracellular signaling via the relatively short ICAM-1 cytoplasmic domain mediates extensive rearrangement of the cytoskeleton in a small Rho-GTPase-dependent manner [82], through the recruitment of multiple actin-binding adapter proteins including cortactin, alpha-actinin, filamen, and ezrin/radixin/moesin protein family members (Figure 3) [83,84,85]. The Rho-GAP protein Deleted in Liver Cancer 1 (DLC-1) and the RhoA-associated guanidine nucleotide exchange factors (GEFs) leukemia-associated Rho GEF (LARG) and epithelial cell transforming sequence 2 (Ect2) have also been implicated in these signaling processes, which are dependent on tractional forces as well as ICAM-1 cross-linking [86,87,88]. While the extracellular domains of ICAM-1 are sufficient for the attachment of leukocytes to the endothelium, in vitro experiments have shown signaling through the cytoplasmic tail to be critical for transendothelial migration of multiple subsets including neutrophils, monocytes, and T cells [84,85,89]. ICAM-1 intracellular signaling via MAPKs p38 and ERK has also been shown to up-regulate expression of multiple pro-inflammatory cytokines including CXCL8, CCL3, CCL4, and COX-2 in human brain microvascular endothelial cells and HUVECs [90,91].

### 3.5. Soluble ICAM-1 Protein

Soluble forms of the ICAM-1 protein (sICAM-1) can be generated by proteolytic cleavage from the cell surface or by translation of an alternative mRNA transcript lacking the transmembrane and cytoplasmic domains [44,92,93,94]. In vitro experiments have demonstrated that increased amounts of sICAM-1 are released from the endothelial cell membrane in response to cytokine stimulation, a process known as ectodomain shedding [92,95]. sICAM-1 contains the five extracellular Ig domains and retains the ability to bind LFA-1 [96]. Elevated sICAM levels have been reported in the serum of patients with numerous conditions, such as sepsis [97] and other infectious diseases including cytomegalovirus [98], latent toxoplasmosis [99], and severe COVID-19 [100], a variety of cancers [101,102,103], and atherosclerosis [104], as well as many systemic inflammatory diseases [105,106,107,108].

In the mouse, all membrane-bound ICAM-1 isoforms appear to be proteolytically cleaved by human leukocyte elastase (HLE) and cathepsin G. Susceptibility varies depending on the enzyme and Ig domain arrangement; for example, isoforms omitting D2 appear the most susceptible to cleavage by cathepsin G [109]. Human in vitro studies have highlighted roles for matrix metalloproteinase (MMP)-9 and closely related a disintegrin and metalloproteinase (ADAM) family members in ectodomain shedding of ICAM-1 [93,109,110]. ADAM17 is implicated in ICAM-1 ectodomain shedding on ICAM-1-expressing HEK293T cells and HUVECs [94], and ADAM10 has been shown to mediate cleavage of ICAM-1 molecules from the cell membrane during neutrophil transmigration of HUVEC monolayers under flow conditions [111].

## 4. Roles of ICAM-1 in Immune Function

ICAM-1 plays key roles in normal immune function, as summarized in this section (Table 1). Perhaps the best studied role is as a central molecular mediator in leukocyte transendothelial migration [19]. While different leukocyte subsets utilize specific mechanisms, in general, the cascade consists of capture, rolling, slow rolling, arrest, firm adhesion, crawling, and diapedesis by either the paracellular or transcellular route. In addition, the involvement of ICAM-1 in immune synapse formation is well established, although the specifics of this involvement remain under investigation. There are emerging roles for ICAM-1 too, such as in wound healing, which continue to expand its known functions.

### 4.1. Leukocyte Trafficking

ICAM-1 binding to LFA-1 and MAC-1 mediates the slow rolling, arrest and firm adhesion, and crawling phases of the transmigration cascade, as well as diapedesis [19]. Conformational changes that activate LFA-1 on the leukocyte surface, mediated in part by ligand binding to endothelial selectins, E-selectin and P-selectin, enhance binding to ICAM-1, slowing leukocyte rolling [112]. Binding of ICAM-1 to activated LFA-1 induces clustering of ICAM-1 and other adhesion molecules in a Rho-dependent manner, facilitating firm adhesion to the endothelium [113]. Crawling at sites of extravasation may be mediated by ICAM-1 interaction with LFA-1 or MAC-1, or both, depending on the leukocyte subset [114,115]. ICAM-1 binding induces the formation of membrane projections rich in F-actin and ICAM-1 clusters that surround the attached leukocytes [83,116], a process that is dependent on numerous Rho family of small GTPases-mediated signaling events [117].

Signaling through the ICAM-1 cytoplasmic domain during the transmigration cascade causes extensive remodeling of the endothelial cell actin cytoskeleton and breakdown of adherens junctions [82,83,84,87,118]. In vitro work with HUVECs and endothelial cells isolated from cord blood demonstrates that neutrophils preferentially migrate via ICAM-1-enriched “hot spots” and these may serve to limit vascular leakage during extravasation [119]. RhoA and ICAM-1-dependent formation of F-actin rich contractile pores at the junctional surface of the endothelium may also limit vascular leakage during leukocyte migration [88]. Studies using in vitro blood–brain barrier models and in vivo live-cell imaging have shown that in conditions of low inflammation, CD4-positive T cells preferentially transmigrate across the endothelium by the paracellular route at ICAM-rich tricellular junctions [120,121,122].

Endothelial cells from different tissues show a high degree of heterogeneity in gene expression across different vascular beds, and between arteries and veins [123]. Molecular differences extend to ICAM-1 expression, which may impact local leukocyte trafficking. We have used transcriptomic and proteomic profiling to show relatively increased basal expression of ICAM-1 by endothelial cells of the human retina compared with those of the choroid [124,125]. In comparison with other Ig superfamily members, ICAM-1 appears to play the major role in trafficking of Th1 cells, Th17 cells, and B cells across human retinal endothelial monolayers in vitro [126,127]. Similar studies of the blood–brain barrier have indicated that ICAM-1 mediates Th1 cell polarization, crawling, and diapedesis through the mouse brain endothelium under non-inflammatory or inflammatory conditions [64]. Using in vivo fluorescence labeling and confocal microscopy, Sumagin et al. showed ICAM-1 expression on activated endothelium could be heterogenous, with higher levels of expression in venules than arterioles in mouse cremaster muscle treated with TNF-α [128]. This difference resulted in an increase in leukocyte firm adhesion to venules but not arterioles.

Early studies of neutrophil migration demonstrated that leukocyte binding to ICAM-1 triggered an increase in endothelial intracellular calcium ion levels and that this change was required for efficient transmigration [129]. Steps in the ICAM-1 signaling cascade that involve remodeling of the actin skeleton and breakdown of endothelial cell adherens junctions have been elucidated, but until recently, the exact mechanism by which endothelial cells potentiate in response to leukocyte binding was unknown [82,83,84,118,130]. A candidate mechanosensitive calcium channel, PIEZO1, has now been uncovered: Wang et al. propose a model where the synergistic activation of ICAM-1 signaling in response to flow forces and leukocyte binding recruits and activates actin-binding adapter proteins, increasing membrane tension and leading to the opening of PIEZO1 [131]. The subsequent increase in intracellular calcium ions drives the activation of Src/Pyk2 tyrosine kinases and ultimately myosin light chain phosphorylation, weakening adherens junction by direct phosphorylation of VE-cadherin and opening the cell–cell junction by actinomyosin contraction.

### 4.2. Immune Synapse

On the surface of antigen-presenting cells, ICAM-1 is involved in the formation of a functional immunological synapse and sustained engagement of the T cell receptor (TCR) with the major histocompatibility complex (MHC)–peptide complex. Work by Grakoui et al. showed real-time development of the immune synapse, a ring-like formation of ICAM-1 and LFA-1 surrounding clustered TCR and MHC–peptide complexes [20]. During the formation of the immune synapse, the actin cytoskeleton of the antigen-presenting cell undergoes extensive remodeling; research suggests the involvement of ICAM-1 signaling in this process, but its role is not yet clear [132]. Zaretsky et al. have demonstrated that the expression of ICAM-1 and ICAM-2 on B cells is required for sustained T cell and B cell engagement and subsequent clonal expansion during the establishment of the germinal center [133].

The indispensability or otherwise of the interaction between ICAM-1 (and ICAM-2 given their functional redundancy) and LFA-1 in T cell priming, proliferation, and differentiation is still being investigated. Scholer et al. demonstrated that a prolonged ICAM-1-mediated interaction between CD8-positive T cells and mature dendritic cells was required for the effective generation of CD8-positive memory T cells but not for their activation and proliferation [134]. Kozlovski et al. and Feigelson et al. used a novel chimeric mouse model to investigate the role of ICAMs in proliferation of naïve T cells in lymph nodes: *Icam2^tm1Jcgr^* mice have an insertion in exon 2 that deletes full-length ICAM-2 [135,136]. These authors transferred hematopoietic cells from wild-type or *Icam1^tm1Jcgr^*/*Icam2^tm1Jcgr^* double-mutant mice into wild-type mice to overcome the defective migration of T cells into lymph nodes observed in fully ICAM-deficient mice and demonstrated that ICAM-1 was dispensable on dendritic cells for naïve CD4-positive T cell proliferation and differentiation in lymph nodes.

### 4.3. Wound Healing

Involvement of ICAM-1 in wound healing partly relates to functions in leukocyte adhesion and trafficking. ICAM-1 is expressed by endothelial cells at the site of skin wounds [21]. In one study, *Icam1^tm1Bay^* mice exhibited impaired wound healing, with relatively few neutrophils and monocytes migrating from the vascular tree into the wound tissue [137]. Another study using *Icam1^null^* mice also showed delayed wound closure in the animals but, surprisingly, no evidence of decreased neutrophil and macrophage infiltration, indicating possible inhibitory effects of alternative ICAM-1 isoforms in *Icam1^tm1Bay^* mice [138]. In other work, intestinal mucosal wounds healed more slowly in *Icam1^tm1Jcgr^* mice, while in wild-type mice, activation of signaling through ICAM-1 with a crosslinking antibody promoted proliferation of intestinal epithelial cells and resulted in faster wound closure [139]. From in vitro experiments, it has been theorized that adhesion of neutrophils via ICAM-1 expressed on the surface of the epithelium activates intracellular signaling through Akt and β-catenin that causes the epithelial cells to proliferate, thus promoting wound healing [139].

ICAM-1 has an emerging role in efferocytosis, the phagocytosis of apoptotic cells by professional phagocytes such as macrophages, which is a critical aspect of the resolution of inflammation and essential for successful wound healing [140]. Wiesolek et al. recently reported that the differentiation of mouse bone marrow-derived macrophages to an inflammatory phenotype ex vivo induced expression of ICAM-1 and increased their ability to phagocytose apoptotic cells in culture, whereas macrophages from *Icam1^tm1Jcgr^* mice displayed impaired phagocytosis [141]. In *Icam1^tm1Jcgr^* mice, macrophages recruited to the peritoneal cavity were less capable of phagocytosing intraperitoneally injected apoptotic intestinal epithelial cells. Antibody-mediated ICAM-1 blockade produced similar effects to those seen in *Icam1^tm1Jcgr^* mice, together indicating ICAM-1 mediates adhesion between macrophages and apoptotic cells during efferocytosis.

In a distinct but related process, which the authors term “enclysis,” Davies et al. studied the engulfment of CD4-positive T cells and preferential destruction of Foxp3-positive regulatory T cells by Huh7 hepatocytic cells and primary hepatocytes [142]. They found that antibody-mediated ICAM-1 blockade inhibited the binding of CD4-positive T cells to hepatocytes and subsequent enclysis, indicating that ICAM-1 is involved in the initiating steps of this process. The process may be an important means of controlling immune responses by regulating T cell subsets and is an interesting avenue for future research.

## 5. Involvement of ICAM-1 in Disease

ICAM-1 has been implicated in the pathophysiological mechanisms underlying a wide range of diseases. Generally, these diseases involve aberrant or excessive activation of inflammatory responses such as occurs in autoimmune diseases or sepsis, and it is clear that the roles of ICAM-1 in adherence, transendothelial migration, and activation of leukocytes could render it both a causative factor in disease and an attractive therapeutic target. Given the range of cell types that express ICAM-1, the various factors that control its expression, splicing, and post-transcriptional and post-translational regulation, as well as the varied signaling pathways in which it is involved, there are abundant context-dependent differences in the roles of ICAM-1 that must be determined for each disease. The following section discusses the known roles of ICAM-1 in several diseases, selected as examples that illustrate the breadth of its involvement across diverse pathology (Table 2).

### 5.1. Sepsis

Sepsis is a life-threatening condition characterized by dysregulated systemic inflammation in response to infection, which can lead to multiple organ failure [160]. Among other adhesion molecules, ICAM-1 is involved in the pathophysiology of sepsis [22]. Although inflammation is necessary for countering infection, excessive systemic inflammation can cause more harm than good. Adhesion molecules such as ICAM-1 facilitate inflammatory reactions that promote dysfunction and breakdown of vascular endothelia, a key pathophysiological step that precipitates organ failure in severe sepsis [161]. The details of the cell types and molecular pathways in which ICAM-1 participates to enhance inflammation in sepsis are not yet fully elucidated but are emerging.

Xu et al. showed that *Icam1^tm1Jcgr^* mice have a large survival advantage over wild-type mice in response to intraperitoneal injection of lipopolysaccharide [59]. Similarly, *Icam1^KO?^* mice have been shown to have a survival advantage in sepsis induced by cecal ligation and puncture compared with wild-type mice [143]. More neutrophils were shown to infiltrate the lung tissue, and plasma concentrations of TNF-α, IL-6, and IL-10 were much higher in wild-type mice than in *Icam1^KO?^* mice. Ode et al. showed that a damage-associated molecular pattern, cold-induced RNA binding protein (CIRP), which is elevated in sepsis, is partly responsible for induction of ICAM-1 on the surface of lung neutrophils following cecal puncture [144]. ICAM-1 positive lung neutrophils exhibit enhanced effector functions, including increased expulsion of neutrophil extracellular traps (NETs), which can induce expression of inflammatory cytokines by macrophages and cause tissue damage [144,162]. Activation of Rho downstream of ICAM-1 leads to the increased expulsion of NETs by neutrophils [162].

Bohatschek et al. showed that intraperitoneal injection of lipopolysaccharide causes an influx of granulocytes into the brain, and subsequent trauma induced by facial axotomy augments their influx into the facial nucleus [145]. Importantly, approximately 50 percent fewer granulocytes were recruited to the facial nucleus in *Icam1^Jcgr^* mice, indicating that ICAM-1 is involved in leukocyte influx into the brain in septic disease [145]. Wang et al. elucidated a mechanism of brain neurovascular damage in the mouse cecal ligation and puncture model of sepsis involving ICAM-1 [163]. Their findings suggest that cecal puncture induces activation of the cation channel P2RX_7_ in brain endothelial cells. Activation of this channel induces the expression of CXCL1 and IL-1β, which together promote ICAM-1 expression and adherence of leukocytes to the brain microvascular endothelium. Leukocyte adherence induces the expression of CX3CL1 by endothelial cells, resulting in chemoattraction and activation of microglia, which are ultimately responsible for damage to the neurovascular tissue surrounding brain microvessels [163].

In addition to these roles of membranous ICAM-1, the circulating sICAM-1 level is higher in septic patients than in healthy individuals [97,164]. Despite much study, the prognostic value of the sICAM-1 level in sepsis is, however, unclear [22]. This is likely in part because shedding of ICAM-1 is not a passive process but enacted by enzymes subject to regulation that may differ between contexts. As well, shedding of adhesion molecules including ICAM-1 may contribute to limiting inflammation by decreasing their density on the endothelium and by enabling the soluble molecules to act as decoy receptors for leukocyte integrins [22]. These factors complexify the relationship between soluble ICAM-1 levels and clinical outcomes in sepsis.

### 5.2. Malaria

In the symptomatic stage of its life cycle in human hosts, the malaria parasite *Plasmodium falciparum* infects erythrocytes [165]. Infected cells can attach to endothelial cells in a process called cytoadherence, which sequesters parasites in the vasculature of essential organs, a key pathological step of severe malarial disease [166,167]. Mechanistically, the parasite expresses a protein called *P. falciparum* erythrocyte membrane protein 1 (PfEMP1), which localizes to the surface of the erythrocyte. PfEMP1 binds to ICAM-1 expressed on the surface of endothelial cells, facilitating cytoadherence of infected cells to the vessel endothelium [17,146]. The site on ICAM-1 bound by PfEMP1 is distinct from that bound by LFA-1 or Mac-1 [168]. ICAM-1 is not the only receptor used by infected erythrocytes to attach to the endothelium; however, ICAM-1 has been found to be highly expressed on the brain endothelial cells of deceased cerebral malaria patients, suggesting its expression may be a key determinant of the severity of cerebral malaria [167].

A polymorphism in *ICAM1*, rs5948 (also known as K469E), has been associated with increased risk of severe malaria in Indian and Nigerian populations [169,170]. This is a single nucleotide change from A to G in the sixth exon of *ICAM1* that results in a coding change from lysine (K) to glutamate (E) at amino acid 469 located in the D5 Ig domain of the ICAM-1 protein. The G allele encoding glutamate has been associated with an increased risk of severe malaria. A mechanism is not immediately clear given PfEMP1 binds a region located in D1 of ICAM-1; the association may reflect a more cryptic immunological change [168]. Another *ICAM1* polymorphism, rs5491 (K29M or ICAM-1^Kilifi^), has been associated with an increased risk of cerebral malaria in Kenyan children and an increased risk of hospitalization due to malaria in Tanzanian children [171,172]. A subsequent study in children from Gabon found the polymorphism was protective, associated with a decreased risk of severe malaria, defined as hyperparasitemia and severe anemia [173]. Studies conducted in other populations have found no association between this polymorphism and the risk of severe and/or cerebral malaria, making the contribution of this polymorphism to the severity of the disease difficult to discern [174,175,176].

### 5.3. Rhinovirus Infections

Human rhinoviruses cause common colds, and in patients with chronic pulmonary diseases, they can exacerbate respiratory symptoms and result in significant and life-threatening morbidity [177]. ICAM-1 is the receptor used by major group rhinoviruses, which encompass over 60% of known strains, for attachment and entry to epithelial cells [16,147,177,178]. The site on ICAM-1 bound by rhinoviruses overlaps that bound by LFA-1 [13]. Monoclonal antibodies to ICAM-1 strongly inhibit the infection of primary human tracheal epithelial cell cultures [148]. Rhinovirus induces expression of IL-1β in these cells, which drives further expression of ICAM-1, and this mechanism likely increases cell infectivity in the early stages of infection [148]. Human airway epithelial cells exhibit low expression of ICAM-1 in their basal state; it is rapidly upregulated during inflammation and in response to rhinovirus [179,180]. It is therefore likely that conditions that elevate ICAM-1 expression confer susceptibility to rhinovirus infection. Strategies to prevent rhinovirus infection have included the use of recombinant soluble ICAM-1 as a decoy receptor able to inhibit infection [181] or the use of a monoclonal anti-ICAM-1 antibody, which blocks binding of rhinovirus to ICAM-1 without preventing the interaction between ICAM-1 and LFA-1 [182].

### 5.4. Multiple Sclerosis

Multiple sclerosis is an autoimmune disease of likely heterogeneous cause in which the formation of inflammatory demyelinating plaques in the central nervous system (CNS) lead to relapsing–remitting or progressive neurological symptoms [183,184]. Myelin-reactive T cells are thought to play a fundamental role in demyelination, but the roles of other cell types including B cells are being discovered.

Experimental autoimmune encephalomyelitis (EAE) is an animal model of multiple sclerosis. It can be induced by injecting mice with specific myelin antigens and adjuvants or by the transfer of myelin-reactive T cells into syngeneic naïve mice. Bullard et al. reported that *Icam1^null^* mice exhibited substantially attenuated EAE [149]. Far fewer leukocytes infiltrated the spinal cord, with almost complete prevention of infiltration of CD4-positive and CD8-positive T cells. Myelin antigen-reactive T cells from wild-type mice fail to induce EAE when transferred into *Icam1^null^* mice, suggesting ICAM-1 expression is required on endothelial cells for the migration of T cells into the CNS in EAE [149]. Moreover, myelin antigen-reactive T cells from *Icam1^null^* mice fail to induce EAE when transferred into wild-type mice, and they exhibit impaired proliferation when stimulated with wild-type antigen presenting cells presenting cognate myelin antigen ex vivo. This suggests ICAM-1 is also required on T cells for the development of EAE.

Deeper inspection of the roles of specific isoforms of ICAM-1 adds complexity to these conclusions. In an earlier study by Samoilova et al., *Icam1^tm1Bay^* mice were found to exhibit more severe EAE than controls, indicating that augmented expression of certain ICAM-1 isoforms exacerbated disease [185]. *Icam1^tm1Jcgr^* mice develop EAE that is attenuated compared with wild-type but more severe than complete *Icam1^null^* mice [186]. From these studies, Bullard et al. reasoned that an isoform lacking the D4 Ig domain was likely responsible for augmented EAE severity [187]. They generated a mouse strain that expressed only this isoform, only on T cells, and found that these mice developed robust EAE after immunization with myelin antigen. Thus, ICAM-1 lacking D4, expressed on T cells, is sufficient to enable EAE development.

Both Th1 and Th17 subsets of CD4-positive T cells are encephalitogenic in EAE. Haghayegh Jahromiet al. used *Icam1^null^*, *Icam2^tm1Jcgr^*, and double-mutant crosses of these to assess the roles of ICAM-1 and ICAM-2 in the migration of Th1 and Th17 cells across the blood–brain barrier into the CNS [65]. They reported that ICAM-1 but not ICAM-2 was required on dendritic cells for activation of cognate CD4-positive T cells in lymph nodes and stimulation of their proliferation. ICAM-1 and ICAM-2 were both found to be necessary on T cells to maintain normal crawling and diapedesis of Th1 and Th17 cells across the blood–brain barrier. Disruption of either ameliorated the EAE severity.

Synthesizing the observations made in mouse models, ICAM-1 is required for the development of EAE through actions both at the immunological synapse, where it is involved in T cell activation, and at the blood–brain barrier, where it facilitates crawling and diapedesis of T cells into the CNS. Whether these observations are informative of equivalent isoform-specific roles of ICAM-1 in human multiple sclerosis remains to be confirmed.

### 5.5. Inflammatory Bowel Disease

Inflammatory bowel disease is a term encompassing two conditions—Crohn’s disease and ulcerative colitis—characterized by pathological inflammation of the gastrointestinal tract, notably the colon [188]. Nielsen et al. reported that the level of serum sICAM-1 is elevated in patients with active inflammatory bowel disease compared with inactive inflammatory bowel disease or controls [189]. The level was higher in patients with Crohn’s disease than ulcerative colitis [189]. Another study found elevated sICAM-1 in patients with active Crohn’s disease compared with controls, and the level decreased with glucocorticoid treatment, although this study did not find higher levels in active than in inactive inflammatory bowel disease [190]. Immunostaining of colonic biopsies from patients with inflammatory bowel disease showed intense ICAM-1 staining of the vascular endothelium, which was weaker or absent in controls [190].

Intraperitoneal administration of anti-ICAM-1 antibody has been shown to greatly reduce tissue damage in a model of colitis induced by dextran sodium sulphate in rats [150]. Far fewer leukocytes invaded the colonic mucosa, suggesting ICAM-1 was required for leukocyte recruitment. Similar results were reported in another study showing that ICAM-1 blockade diminished leukocyte adhesion in colonic venules in a rat model of colitis induced by trinitrobenzene sulfonic acid [151]. In this study, VCAM-1 blockade had a larger effect than ICAM-1 blockade on leukocyte adhesion, indicating ICAM-1 is not the only adhesion molecule with a role in driving inflammation in colitis. *Icam1^tm1Bay^* mice exhibit protection against symptoms, severe lesions, and mortality in dextran sodium sulphate-induced colitis [191,192]. The choice of model appears to matter regarding the involvement of different adhesion molecules. In a model of Crohn’s disease in mice that involves the transfer of CD4-positive T cells from senescence accelerated mouse P-1 (SAMP-1)/Yit mice into severe combined immunodeficiency disease (SCID) mice, antibody blockade of ICAM-1 and VCAM-1 together reduces inflammation, but blockade of either alone does not, suggesting functional redundancy between these two molecules in this model [193].

The inflammatory bowel disease-promoting roles of ICAM-1 must be considered together with the evidence that ICAM-1 is also potentially involved in a protective or resolving capacity. As discussed in Section 4.3, ICAM-1 likely plays a role in healing of wounds to the intestinal mucosa by facilitating neutrophil trafficking and adhesion to the intestinal epithelium [139]. Excessive neutrophil accumulation is a histological characteristic of inflammatory bowel disease, however, so therapeutic modulation of ICAM-1 may need to tread a fine line [194].

### 5.6. Uveitis

ICAM-1 is a player in the pathophysiology of uveitis, a group of intraocular inflammatory diseases that may be caused by a range of infections or have autoimmune or autoinflammatory etiologies [195,196]. sICAM-1 is often elevated in the serum of patients with uveitis, depending on the etiology [197,198,199], and can be predictive of the presence or development of a systemic inflammatory disease [198,200]. The sICAM-1 level in the vitreous is positively associated with uveitis activity and with the level of TNF-α [201].

Lymphocyte access to the retina is usually minimal and is restricted by the blood–retinal barrier, except during infection and inflammation [202]. The blood–retinal barrier is centered at two anatomical sites: the retinal pigment epithelium and the retinal vascular endothelium. ICAM-1 has been shown to be expressed by retinal and choroidal endothelial cells in eyes of human patients with non-infectious uveitis involving the posterior eye in contrast to little or no expression in controls [203]. Molecular profiling studies of human retinal endothelial cell isolates indicate ICAM-1 is relatively highly expressed on this cell population and induced by inflammatory cytokines or microbial products [124,125,204,205]. In vitro, the ability of rat lymphocytes to migrate across monolayers of rat retinal pigment epithelial cells or retinal endothelial cells is inhibited by antibodies against ICAM-1 or LFA-1 [206,207]. Mouse experimental autoimmune uveoretinitis (EAU) is a rodent model of human non-infectious uveitis that is induced by inoculation with specific retinal antigens in complete Freud’s adjuvant; some evidence indicates ICAM-1 may be more prevalent in the retinal pigment epithelium, and VCAM-1 may be more prevalent in the retinal endothelium, suggesting possible site-dependent differences in the processes mediating lymphocyte infiltration in this disease model [208].

The intraperitoneal administration of anti-ICAM-1 or anti-LFA-1 monoclonal antibody completely or partially prevents, respectively, the development of EAU in rats [209]. Analogous results have been reported for endotoxin-induced uveitis, a murine model of anterior uveitis induced by lipopolysaccharide [210]. In this model, antibodies against ICAM-1 or LFA-1 prevent firm adherence and transmigration of lymphocytes into the iris but not rolling of lymphocytes along the vessel surface [211].

ICAM-1 has been implicated in the development of the most common infectious form of uveitis, caused by *Toxoplasma gondii* and often referred to as ocular toxoplasmosis or toxoplasmic retinochoroiditis [212]. Work from our laboratory has revealed that ICAM-1 provides *Toxoplasma gondii* with access to the human retina. Blocking antibody directed against ICAM-1 reduced the numbers of free *T. gondii* tachyzoites that transmigrated human retinal endothelial cell monolayers, and the blockade also limited the passage of *T. gondii*-infected dendritic cells across this cellular barrier [213]. Another group recently reported that a small molecule integrin antagonist, lifitegrast, decreased ocular expression of ICAM-1, CD11a, and CD18 at the protein level and diminished the pathological disruption of the retinal architecture when administered topically to eyes of mice with experimental ocular toxoplasmosis [214].

Although not typically considered an inflammatory disease, one aspect of the retinal pathology of diabetic retinopathy is leukostasis; circulating leukocytes adhere to the retinal vessels, where they are proposed to secrete cytokines that modulate endothelial activation and vessel leakage [215]. ICAM-1, CD11a, CD11b, and CD18 expression are increased in the retina in a streptozotocin-induced model of diabetic retinopathy in the rat [216,217]. Antibody-mediated blockade of ICAM-1 or integrin inhibits leukostasis, and ICAM-1 blockade prevents vascular leakage [216,217], indicating that ICAM-1 likely facilitates leukostasis. It is relevant to note that most evidence for the role of leukostasis in the pathophysiology of diabetic retinopathy has been collected in animal models, and the importance of leukostasis in driving human diabetic retinopathy has been questioned [218].

### 5.7. Cancer

ICAM-1 expression is dysregulated in many cancers, and polymorphisms in the *ICAM1* gene implicate the molecule in cancer susceptibility [219,220,221,222,223,224]. Deciphering the roles of ICAM-1 is an aim of current research in many cancer fields. These roles are not easily summarized; the literature is flush with examples of conflicting data regarding the association of ICAM-1 expression with tumor development, metastasis, and patient prognosis. Depending on the context, ICAM-1 can either benefit a cancer—for example, by facilitating the clustering of circulating tumor cells or acting in pro-tumorigenic signaling pathways—or it can benefit host survival by facilitating the recognition and destruction of tumor cells by the immune system. In most cases, ICAM-1 probably acts for both sides of this fight simultaneously. Whether ICAM-1 expression benefits a cancer more than it benefits the host immune system appears dependent on the cancer type and other contextual factors. The aim of this section is not to provide a definitive explanation of the roles of ICAM-1 in all cancers but to highlight recent studies on a subset of cancers as examples of how ICAM-1 can act to promote cancer, to suppress cancer, or to act as a therapeutic target.

ICAM-1 expression is reportedly elevated in breast cancer cells compared with the surrounding healthy tissue and is expressed especially highly in triple-negative breast cancer tumors [225,226,227]. Knockdown of ICAM-1 in breast cancer cells in vitro has been shown to decrease the expression of genes related to stemness, to decrease invasion of cells through Matrigel, and to decrease transendothelial migration across HUVEC monolayers [152,153]. ICAM-1 may drive metastasis of breast cancer. Homotypic binding between ICAM-1 molecules on breast cancer cells facilitates the formation of circulating tumor cell clusters with high metastatic potential, and antibody blockade of ICAM-1 dramatically decreases lung metastasis without affecting primary tumor growth in a breast cancer cell line xenograft model in mice [153]. Attachment of tumor cells to the endothelium is a key step of metastasis. In another study, ICAM-1-positive breast cancer cells were able to use neutrophil granulocytes as a linker to indirectly attach to endothelial cells in vitro [154]. However, the pro-metastasis view of ICAM-1 is not universally supported. Another article has reported that ICAM-1 expression by breast cancer tumors is associated with longer relapse-free periods and overall survival [155]. These authors utilized a murine luminal B breast cancer cell line, E0771, which is conducive to implantation into immunocompetent C57BL/6 mice and which forms distant metastases. ICAM-1-knockout tumors implanted into mice produced more lung metastases after resection than tumors possessing ICAM-1, which was due to their ability to evade detection by neutrophils in the pulmonary vasculature [155]. Altogether, these results highlight the complexity of the roles of ICAM-1 in its ability to act both in favor of the cancer and of the host.

Expression of membranous ICAM-1 by colorectal tumors has generally been found associated with favorable prognosis [224,228]. Yang et al. reported that ICAM-1 expression is elevated compared with surrounding tissue in colorectal carcinoma, but later disease stages are associated with decreasing expression [156]. They showed that lower ICAM-1 expression permits polarization of macrophages to a tumor-favoring M2 phenotype, increasing the chance of metastasis [156]. It is worth noting that another study found downregulation of ICAM-1 to promote M1 rather than M2 polarization in a different context, so further research is required to identify additional factors that influence macrophage polarization [229]. In contrast to the study above, it has also been reported that ICAM-1 expression positively correlates with colorectal cancer stage [157]. In this study, ICAM-1 expression was shown to favor migration and invasiveness of colorectal cancer cell lines in vitro, and antibody-mediated blockade of ICAM-1 inhibited lung metastasis in a mouse xenograft model [157]. These differing findings are yet to be reconciled.

Commonly, tumor cells are xenografted into highly immunodeficient mice. While these models permit implantation of a wide range of cells and recapitulate many features of normal physiology, the tumor microenvironment will differ from an immunocompetent environment. The effects of interventions targeting ICAM-1 are likely sensitive to the immunological microenvironment, so it is important that results are reported without overextrapolation. A major utility of immunodeficient models, on the other hand, is that they allow discrimination of the functions of ICAM-1 in tumor cells from its functions in the immune system.

ICAM-1 has an important role to play as a facilitator or target of immunological therapies for cancer, especially for hematological malignancies, although it is being increasingly explored as a target in solid tumors as well. Chimeric antigen receptor (CAR) T cells targeting ICAM-1 have been shown to induce temporary remission and extend survival in immunodeficient mouse xenograft models of gastric cancer [230]. In similar xenograft models, ICAM-1 CAR T cells are also able to eliminate thyroid cancer tumors formed by patient-derived advanced thyroid cancer cells [231]. Interestingly, CAR T cells can induce ICAM-1 expression in tumor cells, a feedforward loop that may aid in the destruction of the tumor cells [231,232]. CAR T cells can induce ICAM-1 expression even when not targeted to ICAM-1 [232]. Activation of CAR T cells induces them to release IFN-γ, which can stimulate the expression of ICAM-1 in tumor cells [232]. In addition, ICAM-1 can promote the activation of signaling pathways that induce its own expression. For example, in colorectal cancer cells, it has been shown that ICAM-1 can be phosphorylated on its intracellular domain by c-MET, which enables it to act as an adapter protein between c-MET and Src, promoting signaling [157]. STAT3 is activated downstream and directly upregulates ICAM-1 expression [157]. Intentional upregulation of ICAM-1 with the therapeutic goal of enhancing recognition and killing by CAR T cells, while possibly favorable in terms of cytolytic targeting of tumor cells, must be thoroughly examined in each case given the capacity of ICAM-1 to promote pro-tumorigenic signaling and metastasis.

Finally, ICAM-1 is also potentially a useful marker for the detection and visualization of tumors. Guo et al. showed upregulation of ICAM-1 in triple-negative breast cancers and used iron oxide particles coated with anti-ICAM-1 antibodies to image the tumors in a mouse xenograft model, achieving a signal comparable to previously published human epidermal growth factor receptor 2 (HER2)-targeting probes used for non- triple-negative breast cancer [226].

### 5.8. Cardiovascular Diseases

Cardiovascular diseases are the largest cause of mortality globally and present a major burden on health systems [233]. These disorders include coronary heart disease, peripheral arterial disease, and cerebrovascular disease, among others, which can manifest acutely and devastatingly in the form of stroke, an ischemic limb, or myocardial infarction. While the anatomical sites described by these terms, and the nature of acute symptomatic events, differ, the diseases are most often underpinned by a common pathophysiological process in atherosclerosis [234,235,236,237].

Atherosclerosis is characterized by the development of sites of excessive lipid accumulation in lesions under arterial walls, which progress gradually to form an atherosclerotic plaque with growth of a fibrous cap and necrotic core [238]. Plaques may continue to progress over a period of years, in many cases affecting blood flow and causing ischemia, until a point of rapid disruption causes a serious acute cardiovascular event [238]. Expression of ICAM-1 is elevated in the milieu of atherosclerotic plaques, where it is expressed by endothelial cells, macrophages, and smooth muscle cells; it is generally lowly expressed or absent in surrounding normal artery [239,240,241]. Consistently, this expression co-localizes with areas of leukocyte infiltration [158]. Adherence of monocytes and T cells to endothelial cells and their migration across the endothelium are early events in the development of plaques, consistent with a role for ICAM-1 and other adhesion molecules [25,242,243]. Also of note are the observations that an elevated circulating sICAM-1 level is predictive of the occurrence of carotid artery atherosclerosis and coronary heart disease independent of other risk factors, and the sICAM-1 level correlates with the progression of atherosclerosis in rodent models [244,245,246]. Elevated sICAM-1 associated with early atherosclerosis can be apparent before clinically evident cardiovascular disease develops [104].

The role of ICAM-1 is also suggested by the association of polymorphisms of the *ICAM1* gene with atherosclerosis. These include the K469E polymorphism, but the literature is unclear, in part because of the different ways in which different reports refer to this variant. For example, an early study described it as a C/T transition [247], which is true when considering the antisense strand but unconventional. On the coding strand, it is an A/G transition, the A encoding lysine and the G encoding glutamate. Two more recent meta-analyses appear on the surface to come to opposite conclusions regarding the association of K469E with coronary artery disease [248,249]. One incorrectly states the mutation as an A–G transition, which results in a change from glutamine to lysine [249], and the other, against convention, implies the K469E polymorphism refers to the K-encoding allele rather than the E-encoding allele [248]. Both appear to agree that the A (K) allele is associated with a higher risk and the G (E) allele is associated with a lower risk of coronary artery disease. To avoid confusion, according to Human Genome Variation Society (HGVS) nomenclature, the mutation is NM_000201.3:c.1405A>G (p.Lys469Glu) and, on the basis of these meta-analyses, would be best described as associated with a decreased risk of coronary artery disease among the populations covered by the included studies. A thorough investigation of *ICAM1* gene polymorphisms associated with atherosclerosis is warranted.

Other observations support a role for ICAM-1 in atherosclerosis. ApoE-deficient/*Icam1^tm1Bay^* double-mutant mice have repeatedly been shown to develop less severe aortic lesions with decreased lesion area compared with ApoE-deficient mice [245,250,251]. C57BL/6 mice fed a high-fat and high-cholesterol diet develop atherosclerotic plaques. Nageh et al. showed that *Icam1^tm1Bay^* mice exhibit a 63% decrease in area of atherosclerotic lesions after 20 weeks on a high-fat, high-cholesterol diet compared with wild-type mice [252]. In the same study, knockout of CD18, a β2 integrin subunit, decreased lesion area by 47%, and dual *Icam1^tm1Bay^* mutation and CD18 knockout decreased lesion area by 71% [252]. Another study reported that monocytes adhered to aortic endothelial cells from wild-type but not from *Icam1^tm1Bay^* mice, in response to oxidized low-density lipoprotein [159]. Some results have indicated a role for VCAM-1 in driving atherosclerosis without a requirement for ICAM-1. Nakashima et al. showed that the induction of VCAM-1 expression precedes arterial lesion formation in ApoE-knockout mice, although ICAM-1 is also expressed at plaque-prone sites [242]. Cybulsky et al. observed that the disruption of VCAM-1, but not ICAM-1, decreases plaque area in mice [253]. Their study utilized the *Icam1^tm1Jcgr^* mouse strain; the expression of different sets of ICAM-1 isoforms to the *Icam1^tm1Bay^* strain might contribute to phenotypic differences with the studies discussed above.

It is possible that ICAM-1 expression is sustained in the inflammatory environment of atherosclerotic plaques by continued release of pro-inflammatory cytokines. However, the mechanisms that promote early ICAM-1 expression in arterial endothelial cells are a matter of continued research, although there are some data in this area. For example, ICAM-1 expression may be driven in lesion-prone areas due to differences in the shear forces in the vessels in these areas compared with other areas [242]. A more recent study showed that aldosterone, the level of which correlates with cardiovascular disease severity, induces ICAM-1 expression in arterial endothelial cells in ApoE-deficient mice, and expression is driven largely by activation of the mineralocorticoid receptor [254]. Aldosterone also induces an increase in plaque area, which is abrogated in ApoE-deficient/*Icam1^KO?^* double-mutant mice, showing that ICAM-1 is a key mediator of the pro-atherosclerotic effects of aldosterone.

It has been shown that ICAM-1 present on lipid-membrane microparticles, which are abundant in lesions, can be transferred to the surface of nearby non-activated endothelial cells by fusion of a microparticle membrane with the plasma membrane of an endothelial cell [255]. Exposure of HUVECs to plaque microparticles in vitro increases adhesion of U937 monocytes. The paracrine sharing of adhesion molecules has been suggested as a mechanism promoting plaque progression.

To summarize, there is abundant evidence that ICAM-1 is expressed in atherosclerotic plaques, including at very early stages. There is also strong evidence for a causative role in atherosclerosis, but it has mostly been generated in the *Icam1^tm1Bay^* mouse, with conclusions differing from studies using the *Icam1^tm1Jcgr^* mouse. It would be valuable to replicate these seminal experiments with updated models to solidify understanding of the role of ICAM-1 in atherosclerosis and the progression of cardiovascular disorders.

## 6. Therapeutic Targeting of ICAM-1

Given the major role of ICAM-1 in the development of numerous diseases, particularly inflammatory conditions and cancers, there has been a substantial research effort directed at the development of therapeutics that target the protein. An important consideration in this goal is the role of ICAM-1 in normal physiological processes. Shirani et al. [256] describe “a case in point”: natalizumab, a monoclonal antibody that targets the adhesion molecule on lymphocytes and monocytes, α4-integrin, is associated with multifocal leukoencephalopathy secondary to John Cunningham virus (JCV), attributed to the capacity of the drug to block viral latency. Thus, an intensive systemic blockade of ICAM-1 could be anticipated to have potentially serious adverse effects. Therapeutics directed against ICAM-1 take multiple forms, including blocking monoclonal antibodies, antisense oligonucleotides, CAR T cells, and transcription blockade. In addition to directly targeting ICAM-1 as a mediator of pathology, this adhesion molecule may also be an endothelial marker that directs other therapeutic drugs [257]. Some notable examples are described in this section.

ICAM-1 was the first candidate to be explored in the field of leukocyte trafficking blockade for inflammatory bowel disease [258]. Alicaforsen is an ICAM-1 antisense oligonucleotide that has been studied in multiple clinical trials of inflammatory bowel disease, delivered via several systemic routes [259]. Most recently, an enema-based formulation was investigated for pouchitis, a common complication after surgery for ulcerative colitis, in a phase III randomized controlled clinical trial; although the trial failed to meet primary end-points, there were clinical effects in some patients, and the drug continues under evaluation [260]. Antibodies against ICAM-1 reduce measures of inflammation in rodent models, but this approach has not been taken into human clinical trials [261].

The acute lung injury and acute respiratory distress syndrome spectrum of diseases represents another promising area for ICAM-1 targeting, particularly for directing other therapeutic agents to the pulmonary area. Li et al. conjugated the glucocorticoid, dexamethasone, to anti-ICAM-1 antibody modified as a lipid nanocarrier; they observed reduced severity of lipopolysaccharide-induced mouse lung disease, accompanied by attenuation of leukocyte infiltration and inflammatory cytokine production [262]. Jiang et al. used a different anti-ICAM-1 antibody–lipid nanocarrier conjugate to deliver two anti-inflammatory agents—simvastatin and the angiopoietin-1 gene—in the same model, demonstrating similar effectiveness [263]. Zhang et al. used this system to administer antibiotic and anti-inflammatory drugs—ciprofloxacin and IκB kinase 2 inhibitor, TBCA-1—in a mouse model of acute lung infection with *Pseudomonas aeruginosa*, leading to reductions in the bacterial load and leukocyte extravasation [264]. DNA-based nanocarriers are a new area of investigation in the field of ICAM-1 drug conjugate development [265]. These proof-of-concept studies are yet to be translated to the human setting.

Our research has focused on an alternative method of ICAM-1 blockade in non-infectious uveitis. In vitro human studies show that anti-ICAM-1 antibody prevents Th1 cell, Th17 cell, and B cell migration across the retinal endothelium in a majority of persons [126,127]. Rather than a direct ICAM-1 blockade, we sought to target the induction of the adhesion molecules, thus preserving constitutive ICAM-1 expression. We screened transcription factor candidates that were induced in retinal endothelial cells by inflammatory cytokines [204] and demonstrated C2CD4B or IRF1 blockade could limit ICAM-1 induction on, and leucocyte interactions with, activated retinal endothelium [266].

Almost 30 years ago, anti-ICAM-1 antibody was shown to have activity in a mouse multiple myeloma model [267]. However, a phase I with a follow-up phase II study of the BI-505 monoclonal antibody did not have a therapeutic effect in patients with this malignancy [268,269]. Sherbenou et al. explored the potential in conjugating the antibody with MMAF, a cytotoxic agent [270]. They observed potent and specific myeloma cell cytotoxicity and further showed anti-cancer effects of the conjugate in an orthometastatic myeloma NOD/SCID/γ (NGS) mouse xenograft model.

Several ICAM-1-directed therapeutics have been developed for potential application in solid tumors with poor survival outcomes, such as triple-negative breast cancer. Fukushima et al. used an athymic mouse cancer xenograft model to investigate the effectiveness of near-infrared photoimmunotherapy with an anti-ICAM-1 antibody conjugated to IR700, a silica-phthalocyanine dye that, upon activation by near-infrared light, disrupts cancer cell membranes [271]. The treated mice survived significantly longer than the controls, and tumor growth was slowed. ICAM-1 has also been used to direct multiple drugs to tumor cells [272]. Park et al. and Wei et al. explored opportunity in chimeric antigen receptor (CAR) T cell therapy [225,273]. Park et al. designed a CAR T cell that expressed LFA-1, which they tested in a mouse xenograft model with lung and liver-based 8505 tumor cells, plus metastases [273]. Wei et al. generated an ICAM-1 scFv expressing CAR T cell and studied this in NGS mice with skin tumors [225]. Both groups reported significant reductions in tumor burden in comparison with non-transduced T cells.

The major efforts to target ICAM-1 reflect its key involvement in many major diseases. However, while animal models often have showed positive effects of ICAM-1 blockade overall, there have been disappointing outcomes across multiple human clinical trials, tempered also by the concerns around side effects. Some of the more recent pharmacological advances hold promise for forward movement in this area. Another area of therapeutics is based around using ICAM-1 as a biomarker in drug screening: for example, Gao et al. explored the cardioprotective potential for five-leaf Akebia plant extract by investigating its effect on lipopolysaccharide-induced upregulation of ICAM-1 in human umbilical vein endothelial cells [274].

## 7. Conclusions

ICAM-1 is a molecule that is essential for the normal functioning of the immune system, with roles in leukocyte trafficking and lymphocyte activation, among other established and emerging processes. This also renders it a key player in immune system-mediated pathological processes initiated by infectious diseases or autoimmune reactions. ICAM-1 is therefore an attractive therapeutic target in a wide range of diseases. Given the potential for serious adverse consequences when ICAM-1 is systemically inhibited, there is an ongoing need to better understand the subtleties and complexities of its roles and regulation to inform the development of the next generation of therapeutics.

## Figures and Tables

**Table 1:** Summary of the roles of ICAM-1 in immune processes.

Immune Process	ICAM-1 Role
Leukocyte transendothelial migration	Slow rolling, arrest, and firm adhesion of leukocytes
	Leukocyte crawling and diapedesis
	Assembly of apical membrane projections on endothelium
	Dissociation of adherens junctions in endothelial cells
	Formation of F-actin contractile pores in endothelial cells
Immune synapse formation	Engagement of MHC–peptide complex and T cell receptor
	Clonal expansion of B cells in germinal centers
	Generation of CD8-positive memory T cells
Wound healing	Leukocyte trafficking to wound site
	Epithelial cell proliferation in wound closure
	Efferocytosis
	Enclysis

**Table 2:** Examples of the involvements of ICAM-1 in disease pathogenesis.

Disease	ICAM-1 Involvement	Reference
Sepsis	Leukocyte infiltration into viscera	[143,144,145]
	Release of extracellular traps by neutrophils	
Malaria	Endothelial cytoadherence of parasite infected-erythrocytes	[17,146]
Rhinovirus infection	Attachment of rhinovirus to airway epithelium	[147,148]
Multiple sclerosis	Activation and proliferation of myelin-reactive T cells	[65,149]
	Leukocyte infiltration of the central nervous system	
Inflammatory bowel disease	Leukocyte recruitment via colonic mucosal vasculature	[150,151]
Non-infectious uveitis	Migration of T cells and B cells across retinal endothelium	[126,127]
Cancer	Pro-oncogenic effects: tissue invasion and metastatic trafficking by cancer cells	[152,153,154,155,156,157]
	Anti-oncogenic effects: leukocyte detection of cancer cells	
Cardiovascular disease	Adhesion of T cells and monocytes to arterial endothelium	[158,159]
	Infiltration of leukocytes into atherosclerotic plaques	

## Data Availability

Not applicable.
